# BIRTH WEIGHT AND OVERWEIGHT IN ADOLESCENTS: THE ERICA PROJECT IN THE CITY OF RECIFE, PERNAMBUCO

**DOI:** 10.1590/1984-0462/2021/39/2019380

**Published:** 2021-01-11

**Authors:** Kamilla Brianni de Araújo Gomes, Vanessa Sá Leal, Juliana Souza Oliveira, Crislaine Gonçalves da Silva Pereira, Fabiana Cristina Lima da Silva Pastich Gonçalves, Izabel Siqueira de Andrade, Sophie Helena Eickmann, Pedro Israel Cabral de Lira, Marilia de Carvalho Lima

**Affiliations:** a Universidade Federal de Pernambuco, Recife, PE, Brazil.; b Universidade Federal de Pernambuco, Vitória de Santo Antão, PE, Brazil.

**Keywords:** Birth weight, Adolescence, Obesity, Abdominal obesity, Peso ao nascer, Adolescência, Obesidade, Obesidade abdominal

## Abstract

**Objective:**

To verify the association of anthropometric parameters at birth, socioeconomic and biological variables, physical activity, and parental nutritional status with overweight and abdominal obesity in adolescents.

**Methods:**

A cross-sectional study was carried out on 39 public and private schools in Recife (state of Pernambuco, Brazil). The sample consisted of 1,081 teenagers aged from 12 to 17 years. Data were collected from the Study of Cardiovascular Risks in Adolescents (ERICA). Body mass index according to age (BMI-for-age), waist circumference (WC), and waist-to-height ratio (WtHR) were considered as outcome variables, whereas the explanatory variables were birth weight, Röhrer’s Ponderal Index (RPI), biological and socioeconomic variables, physical activity, and parental nutritional status. The crude and adjusted prevalence ratios (PR) for the studied association were estimated by Poisson Regression.

**Results:**

The multivariate Poisson regression showed that the variable that remained significantly associated with overweight in adolescence was maternal overweight, PR=1.86 (95% confidence interval [95%CI] 1.09-3.17). High birth weight also remained significantly associated with abdominal obesity assessed by WC, PR=3.25 (95%CI 1.0-9.74).

**Conclusions:**

High birth weight may be a marker for abdominal obesity in adolescence; and high maternal BMI, for overweight.

## RESUMO

**Objetivo:**

Verificar a associação de parâmetros antropométricos ao nascer, variáveis socioeconômicas e biológicas, atividade física e estado nutricional parental com excesso de peso e obesidade abdominal de adolescentes.

**Métodos:**

Este estudo transversal foi realizado em 39 escolas públicas e privadas de Recife (PE). A amostra consistiu em 1.081 adolescentes entre 12 e 17 anos de idade, provenientes do Estudo de Riscos Cardiovasculares em Adolescentes (ERICA). Estabeleceram-se como variáveis de desfecho o índice de massa corpórea para a idade (IMC/I), a circunferência da cintura (CC) e a relação cintura/estatura (RCEst), enquanto as explanatórias foram o peso ao nascer, o índice ponderal de Röhrer (IPR), as variáveis biológicas e socioeconômicas, a atividade física e o estado nutricional dos pais. Estimaram-se as razões de prevalência (RP) brutas e ajustadas para as associações estudadas pela regressão de Poisson.

**Resultados:**

A regressão multivariada de Poisson mostrou que a variável mantida como significantemente associada ao excesso de peso na adolescência foi o excesso de peso materno, RP=1,86 (intervalo de confiança de 95% [IC95%] 1,09-3,17). O peso elevado ao nascer também permaneceu bastante associado à obesidade abdominal avaliada pela CC, RP=3,25 (IC95% 1,08-9,74).

**Conclusões:**

O peso elevado ao nascer constituiu marcador para a obesidade abdominal na adolescência; e o IMC materno elevado, para o excesso de peso.

## INTRODUCTION

Over the last decades, overweight has become a worldwide public health issue, with a significant increase in all age groups. In 2013, its prevalence in adolescents aged 12 to 19 years accounted for 23.8 and 22.6% in boys and girls, respectively, from developed countries; and 12.9 and 13.4%, in developing countries.^1^

The period of greatest risk for the incidence of obesity is the transition between adolescence and adulthood, due to the greater vulnerability resulting from physiological and psychosocial changes inherent in this stage.^2^ The literature reports that comorbidities, such as insulin resistance, arterial hypertension, and dyslipidemia, can arise from childhood and adolescence, compromising the quality of life and increasing the risk of death in later stages.^2^

The search for understanding how multiple elements can make individuals susceptible to the development of chronic noncommunicable diseases (NCDs) has been the basis for clinical and epidemiological studies, from the investigation of gene expression to environmental factors that can act even in the intrauterine period.^3^ Researchers associate birth weight with obesity and other NCDs in adulthood, in such a way that being born with low or high weight seems to influence future nutritional status.^4^

About eight decades ago, studies were carried out in order to investigate possible associations between characteristics of fetal development and the individual’s future health conditions.^3^ Henceforth, concepts that suggest mechanisms by which an inadequate intrauterine environment can influence the risk of NCDs in adulthood have been formulated. Therefore, some hypotheses were formulated, such as Barker’s, which suggested an association between inadequate fetal nutrition and cardiovascular diseases; the thrifty phenotype hypothesis, which associates nutritional deficiency with characteristics of an organism with low energy expenditure; in addition to other more current theories, such as “predictive adaptive responses” and “maternal capital,” which are closer to the concepts of phenotypic and epigenetic plasticity.^3^ ^,^ ^5^

Among other factors deemed obesogenic, the literature also points out sociodemographic aspects,^6^ the alterable environmental factors that contribute to the positive energy balance, such as high dietary intake and physical inactivity,^7^ in addition to parental overweight.^8^ Considering the multifactorial genesis of obesity and its repercussions on the population’s health, several studies have been published in recent decades in order to understand the physiological behavior of the disease in different population groups, especially in childhood and adolescence, in which the emergence of chronic diseases can become persistent.

Taking this into consideration, this study aimed at verifying the association of anthropometric parameters at birth, socioeconomic and biological variables, physical activity, and parental nutritional status with overweight and abdominal obesity in adolescents.

## METHOD

This is a cross-sectional study carried out with adolescents aged 12 to 17 years, in accordance with the concept of adolescence established by the Child and Adolescent Statute,^9^ of both sexes, enrolled in public and private schools in the municipality of Recife, state of Pernambuco, Brazil. Data were obtained from a national multicenter school-based study entitled “Study of Cardiovascular Risks in Adolescents” (*Estudo de Riscos Cardiovasculares em Adolescentes* - ERICA), whose objective was to estimate the prevalence of cardiovascular risk factors and metabolic changes in the studied sample. Data collection was carried out in schools between October 2013 and May 2014.

According to the adopted eligibility criteria, individuals with physical disabilities that prevented the anthropometric assessment, pregnant adolescents, and those with endogenous obesity or obesity of secondary cause were excluded. Students enrolled in the morning and afternoon shifts, in classes from the 7^th^ to the 9^th^ grade of Elementary School and from the 1^st^ to the 3^rd^ grade of High School were included, since, considering students without a school gap, the age group of 12 to 17 years is expected to be enrolled in the eligible grades.^10^

The national population of ERICA was segmented into 32 geographic strata, consisting of 27 capitals and 5 sets of cities with more than 100 thousand inhabitants. The sampling process, detailed by Vasconcellos et al.,^10^ included probabilistic selection of schools, combinations of shifts, grades, and classes, in such a way that the distribution of schools per situation (urban or rural) and administrative dependence (public or private) in each stratum was sought to be maintained. When there was refusal to participate, the school was replaced by another with similar characteristics. To compensate for expected losses related to the lack of response and others, the sample size was increased by 15%.

The research results have national representativeness for all strata and macro-regions of the country. For this study, a representative sample for the city of Recife was used, collected in 39 schools and composed of 1,081 adolescents.

Questionnaires were applied to the students, which were self-administered in an electronic data collector, the Personal Digital Assistant (PDA); and to their guardians, through a printed form that was sent to them, whose contents comprised personal, socioeconomic, demographic, nutritional, and behavioral information. Anthropometric variables were collected by trained researchers, registered on the PDA (as in the case of the questionnaire), and sent to the ERICA central server.^11^

The collection of anthropometric information at birth took place through a questionnaire responded by the adolescents’ guardians. Birth indicators were deemed as exposure variables, namely: weight and Röhrer’s Ponderal Index (RPI), a measure used to assess body proportionality. Weight was categorized as low/inadequate (<3000 g), adequate (3000 to 3999 g), and high (≥4000 g).^12^ This categorization was due to the sample size, because, separately, the portion of the study population with low birth weight (<2500 g) only accounted for 59 individuals. In order to make a statistical analysis with a more expressive sample feasible, it was decided to gather both groups into a single category, totaling 204 individuals. RPI (weight[g]/length[cm]^3^ × 100) classified the individuals as proportional, ≥2.5 g/cm^3^, and disproportionate, <2.5 g/cm^3^.^13^ Students were qualified according to the gestation period in preterm, less than nine months, and full-term (period equal to or greater than nine months).

The method used for gauging anthropometric measurements was described by Bloch et al.^11^ The adolescents’ body weight and height determined the body mass index according to age (BMI-for-age) assessed by the AnthroPlus software (2007), and measurements of waist circumference (WC) and height were part of the calculation of the waist-to-height ratio (WtHR). The following cutoff points were adopted: BMI-for-age^14^ score Z≥+1 for overweight; WC≥90th percentile (P90)-^15^, and WtHR≥0.50^16^ for abdominal obesity.

Two parameters were chosen to assess abdominal fat, considering that WC has an important correlation with imaging tests deemed the gold standard for estimating abdominal fat;^17^ however, for some authors, WtHR is considered as the best index for predicting risk factors in children and adolescents, among other anthropometric measures.^18^

Socioeconomic profile was evaluated so that individuals were categorized according to the type of school they attended (public or private), maternal education (education time ≤8 and ≥9 years of study), and socioeconomic class. This last variable met the Brazilian Economic Classification Criteria, proposed by *Associação Brasileira de Empresas de Pesquisa* [Brazilian Association of Research Companies].^19^ For this study, the subcategories of classes A1, A2, B1, and B2 were grouped into upper class; and C, D, and E, into low class.

Age was categorized into two age groups, based on the median of 14 years of age: 12 to 14 years, and 15 to 17 years. The sexual maturation stage was self-reported and established according to Tanner’s criteria,^20^ classified as follows: pubertal (stages I, II, III, and IV) and postpubertal (stage V).

The variable obtained for assessing lifestyle was physical activity, whose level was determined in accordance with the *International Physical Activity Questionnaire* (IPAQ).^21^ Adolescents who did not perform any leisure-time physical activity or performed less than 300 minutes of physical activity per week were considered inactive; those who did some leisure-time physical activity with a workload of 300 to 2,100 min/week were considered active. Students whose workload of leisure-time physical activity exceeded 2,100 minutes per week were considered missing for this variable (measurement error), and totaled 64 adolescents. Moreover, paternal and maternal BMI were considered as covariates, according to the World Health Organization (WHO)^22^ classification for overweight (BMI ≥25 kg/m^2^). To obtain the BMI, measures mentioned by the parents during the data collection period were used.

Considering the complex sampling design of ERICA, statistical analyses were performed with adjustment of the survey module, in the STATA software version 13.0. In order to investigate the association of explanatory variables of the model with BMI-for-age, WC, and WtHR, bivariate analyses were initially performed using the simple Poisson regression. Subsequently, a hierarchical model was adopted, in which independent variables were grouped into three levels of influence in determining overweight and abdominal obesity assessed by waist circumference, in accordance with evidence in the literature.

This model was considered for multivariate regression analysis, with robust adjustment of the variance, for which variables with p≤0.20 in the bivariate analysis were selected. Variables of the first hierarchical level were analyzed and, successively, those of subsequent levels were included in the model, without disregarding the previously analyzed levels. At the end of the model, only values of p<0.05 were considered statistically significant. Results were presented in crude and adjusted prevalence ratios (PR), with a 95% confidence interval (95%CI).

This study was approved by the Research Ethics Committee of Universidade Federal de Pernambuco, under CAAE registration number: 05185212.2.2002.5208. All participants signed an informed consent form.

## RESULTS

The anthropometric, biological, socioeconomic, physical activity-related, and parental characteristics of the 1,081 adolescents are demonstrated in Table 1. Fewer responses were verified for the variables birth weight (857), RPI (696), paternal BMI (611), and maternal BMI (758).

**Table 1:** Characterization of the sample according to nutritional status in adolescence and at birth, biological, socioeconomic, physical activity-related, and parental variables of adolescents (n=1,081). Recife, 2013-2014.

	n	%	95%CI
Sex			
Female	659	49.6	-
Male	422	50.4	-
Age			
12-14	564	50.9	-
15-17	517	49.1	-
BMI-for-age			
Without overweight	775	70.9	67.2-74.3
Overweight	306	29.1	25.6-32.7
WC			
Without abdominal obesity	972	89.1	86.1-91.4
Abdominal obesity	108	10.9	8.5-13.8
Waist-to-height ratio			
Without abdominal obesity	923	85.3	82.4-87.7
Abdominal obesity	157	14.7	12.2-17.5
Birth weight			
Low/inadequate (<3000 g)	204	21.4	18.1-25.0
Adequate (≥3000 to 3999 g)	564	66.7	62.9-70.1
High (≥4000 g)	89	11.9	9.8-14.3
RPI (g/cm^3^)			
Disproportionate	196	28.2	24.0-32.6
Proportional	502	71.8	67.3-75.9
Sexual maturation			
Pubertal	741	67.6	63.5-71.4
Postpubertal	338	32.4	28.5-36.4
Gestation period			
Preterm	94	10.3	0.7-13.7
Full-term	848	89.7	86.2-92.3
Maternal education (years)			
≤8	231	29.2	20.7-39.3
≥9	597	70.8	60.6-79.2
Economic class			
Upper	447	58.8	48.8-68.1
Low	318	41.2	31.8-51.1
Type of school			
Public	674	60.1	40.4-76.9
Private	407	39.9	23.0-59.5
Physical activity			
Inactive	495	45.1	39.8-50.4
Active	522	54.9	49.5-60.1
Paternal BMI			
Without overweight	178	27.9	22.8-33.6
Overweight	433	72.1	66.3-77.1
Maternal BMI			
Without overweight	315	44.5	39.9-49.0
Overweight	443	55.5	50.9-60.0

BMI: body mass index; WC: waist circumference; WtHR: waist-to-height ratio; RPI: Röhrer’s Ponderal Index; 95%CI: 95% confidence interval.

The distribution of overweight according to explanatory variables is demonstrated in Table 2. It is observed that high birth weight is associated with a higher prevalence of overweight in adolescence, PR=1.63 (95%CI 1.16-2.29). Among biological factors, male sex and the age group of 12 to 14 years indicated a significantly higher BMI-for-age. Children of overweight or obese mothers have almost twice the prevalence of overweight in relation to adolescents whose mothers do not have these characteristics, PR=1.99 (95%CI 1.29-3.08).

**Table 2:** Prevalence of overweight in adolescents according to nutritional status at birth, biological, lifestyle, socioeconomic, and parental variables (n = 1,081). Recife, 2013-2014.

	Overweight (BMI-for-age)				
	n_observed_	n_estimated_	%	PR (95%CI)	p-value
At birth					
Weight					
Low/inadequate (<3000 g)	51	6,369	30.0	1.07 (0.64-1.78)	0.777
Adequate (≥3000 to 3999 g)	162	18,584	28.1	1.0	
High (≥4000 g)	39	5,426	45.8	1.63 (1.16-2.29)	0.006
RPI (g/cm^3^)					
Disproportionate (<2.5)	50	6,562	23.5	1.0	
Proportional (≥2.5)	153	23,436	32.9	1.38 (0.87-2.19)	0.157
Gestation period					
Preterm	30	4,381	42.8	1.53 (0.89-2.63)	0.115
Full-term	239	25,187	28.3	1.0	
Sex					
Female	172	12,273	24.9	1.0	
Male	134	16,562	33.1	1.32 (1.04-1.68)	0.022
In adolescence					
Age (years)					
12-14	177	16,959	33.6	1.37 (1.06-1.79)	0.018
15-17	129	11,876	24.3	1.0	
Sexual maturation					
Pubertal	194	19,187	28.6	1.0	
Postpubertal	111	9,595	29.9	1.04 (0.78-1.38)	0.755
Physical activity					
Inactive	128	12,855	28.7	0.97 (0.70-1.33)	0.860
Active	160	16,070	29.5	1.0	
Socioeconomic factors					
Maternal education (years)					
≤8	70	10,510	36.3	1.0	
≥9	170	19,296	27.4	0.98 (0.87-1.09)	0.718
Economic class					
Upper	129	15,822	27.1	1.0	
Low	91	13,362	32.7	1.14 (0.71-1.82)	0.551
Type of school					
Public	182	15,867	26.6	1.0	
Private	124	12,967	32.8	1.23 (0.93-1.62)	0.130
Parental nutritional status					
Paternal BMI					
Without overweight	44	6,072	21.9	1.0	
Overweight	132	23,468	32.8	1.45 (0.72-2.94)	0.280
Maternal BMI					
Without overweight	62	8,577	19.4	1.0	
Overweight	156	21,724	39.4	1.99 (1.29-3.08)	0.003

BMI: body mass index; RPI: Röhrer’s Ponderal Index; 95%CI: 95% confidence interval; PR: prevalence ratio.

According to WC, abdominal obesity was 2.8 times more prevalent, PR=2.8 (95%CI 1.14-6.87), in individuals who were born with high weight in relation to those who were born with weight lower than 3000 g, as demonstrated in Table 3. The association of maternal BMI with WtHR indicated a twice higher prevalence of abdominal obesity in children of mothers with BMI higher than 25 kg/m^2^, PR=2.05 (95%CI 1.07 -3.93), as observed in Table 4.

**Table 3:** Prevalence of high waist circumference in adolescents, according to nutritional status at birth, biological, lifestyle, socioeconomic, and parental variables (n=1,081). Recife, 2013-2014.

	Abdominal obesity (WC)				
	n_observed_	n_estimated_	%	PR (95%CI)	p-value
At birth					
Weight					
Low/inadequate (<3000 g)	14	1,749	8.2	1.0	
Adequate (≥3000 to 3999 g)	55	6,882	10.4	1.31 (0.58-2.95)	0.498
High (≥4000 g)	16	2,574	21.7	2.80 (1.14-6.87)	0.026
RPI (g/cm^3^)					
Disproportionate (<2.5)	20	3,257	11.6	1.10 (0.48-2.54)	0.802
Proportional (≥2.5)	49	7,517	10.5	1.0	
Gestation period					
Preterm	9	1,438	14.0	1.34 (0.49-3.63)	0.550
Full-term	85	9,509	10.7	1.0	
Sex					
Female	58	4,704	9.5	1.0	
Male	50	6,136	12.2	1.28 (0.83-1.98)	0.872
In adolescence					
Age (years)					
12-14	39	4,366	8.6	1.0	
15-17	69	6,474	13.3	1.53 (0.92-2.55)	0.095
Sexual maturation					
Pubertal	63	7,149	10.6	1.0	
Postpubertal	45	3,704	11.5	1.07 (0.70-1.65)	0.717
Physical activity					
Inactive	46	4,587	10.3	1.0	
Active	56	6,318	11.6	1.12 (0.67-1.90)	0.635
Socioeconomic factors					
Maternal education (years)					
≤8	22	2,752	9.5	1.0	
≥9	63	7,097	10.1	1.04 (0.86-1.27)	0.627
Economic class					
Upper	42	5,160	8.8	1.0	
Low	40	5,025	12.3	1.34 (0.63-2.86)	0.423
Type of school					
Public	66	6,282	10.5	1.0	
Private	42	4,558	11.5	1.09 (0.70-1.70)	0.679
Parental nutritional status					
Paternal BMI					
Without overweight	13	1,523	5.5	1.0	
Overweight	49	9,834	13.7	2.44 (0.62-9.56)	0.191
Maternal BMI					
Without overweight	15	2,959	6.7	1.0	
Overweight	57	7,999	14.5	2.20 (0.84-5.75)	0.103

WC: Waist circumference; RPI: Röhrer’s Ponderal Index; BMI: body mass index; 95%CI: 95% confidence interval; PR: prevalence ratio.

**Table 4:** Prevalence of high waist-to-height ratio in adolescents according to nutritional status at birth, biological, lifestyle, socioeconomic, and parental variables (n=1,081). Recife, 2013-2014.

	Abdominal obesity (WtHR)				
	n_observed_	n_estimated_	%	PR (95%CI)	p-value
At birth					
Weight					
Low/inadequate (<3000 g)	28	2,811	13.2	1.0	0.726
Adequate (≥3000 to 3999 g)	81	9,564	14.5	1.11 (0.59-2.10)	
High (≥4000 g)	18	2,788	23.5	1.84 (0.76-4.41)	0.246
RPI (g/cm^3^)					
Disproportionate (<2.5)	30	4,734	16.9	1.17 (0.57-2.41)	0.651
Proportional (≥2.5)	73	10,044	14.1	1.0	
Gestation period					
Preterm	18	2,158	21.1	1.48 (0.73-2.99)	0.253
Full-term	121	12,830	14.4	1.0	
Sex					
Female	98	7,429	15.1	1.05 (0.74-1.50)	0.757
Male	59	7,161	14.3	1.0	
In adolescence					
Age (years)					
12-14	79	7,660	15.2	1.06 (0.74-1.52)	0.708
15-17	78	6,930	14.2	1.0	
Sexual maturation					
Pubertal	98	9,833	14.6	1.0	0.952
Postpubertal	59	4,771	14.8	1.01 (0.69-1.46)	
Physical activity					
Inactive	75	6,765	15.1	1.02 (0.70-1.49)	0.896
Active	74	8,035	14.7	1.0	
Socioeconomic factors					
Maternal education (years)					
≤8	36	4,396	15.2	1.0	
≥9	86	9,299	13.2	1.0	
Economic class					
Upper	65	7,585	12.9	1.0	
Low	53	6,711	16.4	1.25 (0.67-2.34)	0.454
Type of school					
Public	98	8,819	14.7	1.0	
Private	59	5,771	14.6	1.01 (0.68-1.49)	0.954
Parental nutritional status					
Paternal BMI					
Without overweight	21	2,678	9.6	1.0	
Overweight	69	12,818	17.9	1.77 (0.47-6.56)	0.379
Maternal BMI					
Without overweight	24	4,202	9.4	1.0	
Overweight	83	10,654	19.4	2.05 (1.07-3.93)	0.031

RPI: Röhrer’s Ponderal Index; BMI: body mass index; 95%CI: 95% confidence interval; PR: prevalence ratio.

The results of the multiple Poisson regression for the outcomes BMI-for-age and WC indicated that the explanatory variable “maternal overweight” remained significantly associated with overweight (PR=1.86; 95%CI 1.09-3.17; p=0.024). Adolescents who were born weighing ≥4000 g remained with a higher prevalence of abdominal obesity assessed by WC at the end of the model (PR=3.25; 95%CI 1.08-9.74; p=0.036).

## DISCUSSION

This research evaluated the nutritional status of a representative sample of adolescents from public and private schools in the city of Recife, aiming at assessing the influence of anthropometric parameters at birth on overweight and abdominal obesity in adolescence. About a third of the adolescents were overweight, according to BMI, and had lower proportions of abdominal obesity, as verified by WC and WtHR. Adolescents who were born with 4000 g or more had a higher prevalence of abdominal obesity, and those whose mothers had a high BMI showed a higher proportion of overweight.

The prevalence of overweight and abdominal obesity assessed by WC were higher than the findings of another study carried out in Pernambuco, in the municipality of Vitória de Santo Antão, Brazil, in which the same cutoff points were used for the outcomes BMI-for-age, WC, and WtHR. A 17.8% proportion of students aged 10 to 19 years were overweight, and 4.2% had abdominal obesity according to WC. As for WtHR, 11.4% of the adolescents presented higher values, similar to the present study.^23^ The prevalence of overweight is higher than that found for abdominal obesity measures, which suggests that BMI only detects the growth of body mass, and not the concentration of fat in specific regions.^6^

In the present study, adolescents who were born with high weight demonstrated a higher prevalence of overweight and abdominal obesity assessed by WC; however, after the statistical adjustments of the multivariate analysis, only the association with WC remained significant. This result may suggest a greater influence of other variables and other growth stages in the determination of BMI, whereas birth weight would have a greater influence on the distribution of body fat.

Low or inadequate birth weight (<3000 g) was not associated with nutritional status in adolescence in the present investigation. Nevertheless, some factors may have contributed to this finding, such as the small number of children with low weight in the studied sample and the categorization of birth weight, considering that this was different from the one established by a study that identified higher body weight and abdominal fat in children who were born with low weight.^24^

Some metabolic adjustments can be precociously structured so that the organism survives under postnatal conditions expected in the prenatal environment - for instance, when there is a restriction in the supply of nutrients to the fetus. Some hypotheses suggest that, in this situation, the use of maternal supplies for the development of vital organs is given priority over other tissues. Fetal hypoglycemia occurs, stimulating protein catabolism, and there is a reduction in the insulin-like growth factor (IGF-1). These conditions would compromise the growth of muscle mass, promoting less metabolic activity and insulin resistance, and would be associated with the development of overweight in individuals who were born with low weight.^3^ ^,^ ^4^

Other factors can intervene in the long-term development of illnesses for individuals who were born with reduced weight. Bernardi et al.,^25^ in a cohort study carried out between 1978/1979 and 2002/2004 in the city of Ribeirão Preto, São Paulo, Brazil, investigated the influence of the concept of social mobility on metabolic characteristics of adult individuals. The results indicated that, among women who were born with low weight, BMI and WC were significantly higher for those who did not show improvement in socioeconomic conditions throughout life.

These data demonstrate that social and environmental changes can alter the prognosis indicated by some hypotheses related to the origin of diseases. Biologically, the foundation for the association between the individual’s socioeconomic mobility and health may be related to changes in stress mechanisms and, consequently, in the cardiovascular system, considering that it is one of the most vulnerable systems in this regard.^25^

It was not possible to assess the body proportionality of 36% of the sample, which probably explains the lack of association between RPI and anthropometric parameters in adolescence. The analysis of this variable is important to indicate the moment when intrauterine nutritional deprivation may have occurred. Proportional adolescents (RPI≥2.5) may have presented linear growth impairment at birth, which suggests nutritional restrictions at the beginning of pregnancy, when there is greater cell differentiation and the formation of hypothalamus and vital organs, making the individual more vulnerable to the development of overweight.^4^

There was no significant association between socioeconomic variables and the studied outcomes. One of the possible reasons reported by Vasconcellos et al.^26^ for the lack of association with maternal education is related to the abundance of information disseminated by virtual media and television, for instance, to which adolescents have access, making the parents’ role in the process of obesity prevention less relevant.

Similar to socioeconomic status, there was also no association between physical activity and outcome variables in the present study. Although physical activity did not influence nutritional status, it is noteworthy that the number of active individuals was slightly higher in the studied sample. Despite being a widely employed method, with validation for the Brazilian population and of low cost, the use of IPAQ has some limitations that may have influenced the results. The perception of the intensity of each activity and the difficulty in measuring the duration of such activities are emphasized, especially those that considerably vary on a day-to-day basis such as activities carried out in the domestic environment.

Maternal BMI was significantly associated with overweight in the crude and adjusted analyses, and with abdominal obesity assessed by the WtHR. These results are similar to those of another study carried out in the state of Pernambuco, whose sample of 1,435 children and adolescents aged 5 to 19 years indicated that the occurrence of overweight among children of mothers with this diagnosis was twice as likely.^27^ Such results can be explained by the role of genetic factors, in addition to socio-environmental conditions, since parents exert influence over food choices, performance of physical activity, and the adoption of sedentary behavior on the part of children and adolescents.^28^

Despite the lack of statistical significance between associations regarding maternal BMI and WC, as well as the paternal BMI and the three outcome variables, it is worth highlighting the width of the confidence intervals, which may suggest the need for increasing the sample to verify significant results.

This study has limitations that have reduced its ability to demonstrate some significant associations, if any. The cross-sectional design is one of them, which limits the determination of cause-and-effect relationships, in addition to the lack of data regarding anthropometric variables at birth and parental nutritional status. Other variables could improve the investigation, providing information on triggering factors of low or high birth weight and on nutritional aspects that influence the development of overweight, for example: maternal information prior to pregnancy, such as nutritional status and dietary factors; monitoring of postnatal weight gain, and data on the child’s complementary feeding.

Furthermore, it is worth emphasizing that the sample calculation and data collection of the present cases were not primarily directed to the investigation of neonatal parameters, which have been mentioned by the guardians and, thus, are subject to memory bias.

In conclusion, high birth weight influenced the onset of abdominal obesity in the studied population, and maternal nutritional status consisted in a relevant factor in determining overweight in adolescents.
